# The Present Attitude of Veterinarians on the Subject of Tuberculosis

**Published:** 1890-03

**Authors:** Daniel D. Lee

**Affiliations:** Instructor of Anatomy, Vet. Dep., Harvard University


### THE PRESENT ATTITUDE OF VETERINARIANS ON THE SUBJECT OF TUBERCULOSIS.

By Daniel D. Lee, M.D.V.,
*Instructor of Anatomy, Vet. Dep., Harvard University.*

All veterinarians who have had the advantage of a modern education in their profession are thoroughly convinced that tuberculosis is a contagious disease.

They are aware that the disease can be produced by the inoculation of milk, of muscle juice or tuberculous products from animals suffering from the disease, and also that it is transmitted from animal to animal and from man to animals by cohabitation and inhalation of dust containing dried sputa.

Many veterinarians insist on the slaughter of all suspected animals and at the same time forbid the use of their milk or meat for food.

Infection can undoubtedly be produced by *constantly* feeding animals with tuberculous milk or meat, but even so enthusiastic an authority as Prof. Arloing acknowledges that this is the *least* important source of contagion.

We certainly do not use such products constantly for our daily food, for the number of cattle found tuberculous averages about 5 in 1000.

The chief source of danger, both for animals and men, lies in the inhalation of dust containing the dried sputa in those localities where the disease is prevalent and the population dense.

Cattle kept in such localities for milk, in badly ventilated barns, are diseased sometimes as high as 40 per cent. to 50 per cent., and are certainly as liable to contagion from tuberculous human beings as from one of their own kind.

Under these circumstances, even if we kill off all the tuberculous animals, shall we have accomplished anything?

Will not cows, pigs and chickens coming into contact with tuberculous human beings, eating their sputa and breathing it in the form of dust, again contract the disease? Pet dogs and cats have done so.

In my opinion it is waste of time, and sometimes unjustifiable destruction of property, to kill off all our tuberculous animals, *unless* some means are adopted by the *medical profession* to quarantine their own patients.

In this part of the country people are beginning to have a horror of a tuberculous cow or pig, but feel not the slightest fear from direct contact with a person in an advanced stage of pulmonary tuberculosis, living in the same room, eating from the same dishes, and freely inhaling their dried sputa.

Nor did I ever know of a case where precautions were taken against the infection of cattle or other animals from tuberculous human beings.

Now, as cattle are especially susceptible to tuberculosis, and as they have already greatly benefited the human race by furnishing it with the means of lessening the ravages of the small-pox, and as cattle are affected with tuberculosis in the largest percentage where they are kept for milk in localities where the percentage of the disease is also very great, I enter a plea that the severity of the crusade against them be somewhat lessened, until some steps are taken by the medical profession and boards of health to quarantine human beings suffering from tuberculosis.

I wish it understood that I believe tuberculosis to be a very contagious disease, but slow in its course.

Everyone will acknowledge that the danger from the milk and meat is the *very least.* The milk is diluted by that of healthy cows, under which circumstances even direct inoculation often fails, and the meat is only diseased in five cases in 1000, and then is generally cooked.

The danger from inhalation of dried sputa in the dust is very great either from man to man, or man to animals.

Therefore, let us wait a little before we condemn all the cattle and other diseased animals, for even if we eradicate the disease among them themselves they will contract it again from man.

